# Methane excess in Arctic surface water- triggered by sea ice formation and melting

**DOI:** 10.1038/srep16179

**Published:** 2015-11-10

**Authors:** E. Damm, B. Rudels, U. Schauer, S. Mau, G. Dieckmann

**Affiliations:** 1 Alfred Wegener Institute Helmholtz-Centre for Polar and Marine Research P.O. Box 12061, 27515 Bremerhaven, Germany; 2 Finnish Meteorological Institute, Erik Palmenin aukio 1 P.O. Box 503, FI-00101 Helsinki, Finland; 3 University of Bremen, Section Geoscience, P.O. Box 330440, 28334 Bremen, Germany

## Abstract

Arctic amplification of global warming has led to increased summer sea ice retreat, which influences gas exchange between the Arctic Ocean and the atmosphere where sea ice previously acted as a physical barrier. Indeed, recently observed enhanced atmospheric methane concentrations in Arctic regions with fractional sea-ice cover point to unexpected feedbacks in cycling of methane. We report on methane excess in sea ice-influenced water masses in the interior Arctic Ocean and provide evidence that sea ice is a potential source. We show that methane release from sea ice into the ocean occurs via brine drainage during freezing and melting i.e. in winter and spring. In summer under a fractional sea ice cover, reduced turbulence restricts gas transfer, then seawater acts as buffer in which methane remains entrained. However, in autumn and winter surface convection initiates pronounced efflux of methane from the ice covered ocean to the atmosphere. Our results demonstrate that sea ice-sourced methane cycles seasonally between sea ice, sea-ice-influenced seawater and the atmosphere, while the deeper ocean remains decoupled. Freshening due to summer sea ice retreat will enhance this decoupling, which restricts the capacity of the deeper Arctic Ocean to act as a sink for this greenhouse gas.

The Arctic is considered a region where noticeable and rapid global change is taking place and where the effects, particularly on sea ice, are dramatic, albeit that the area is relatively small globally seen^1^,^2^,^3^,^4^. Summer sea ice retreat increases exposure of surface water, thus enhancing gas exchange between the Arctic Ocean and the atmosphere where formerly sea ice acted as a physical barrier for sea air flux^5^,^6^. Also gas transfer in the opposite direction is inhibited as ocean freshening due sea ice retreat isolates the sea water below the halocline from the atmosphere^7^. Elevated atmospheric methane concentrations over open leads and regions with fractional sea-ice cover in the remote Arctic^8^ indeed reveal feedbacks as yet unaccounted for in biogeochemical cycling of methane between sea ice, sea-ice influenced water and the atmosphere. Methane super-saturation recently detected in the vicinity of marginal ice zones, under multiyear sea ice and in polynyas^9^,^10^,^11^ also point to pathways of methane discharge in a partly ice covered ocean and focuses the view on a specific sea ice- ocean- atmosphere coupling in addition to the known direct sea ice-atmosphere efflux. The fact that sea ice itself constitutes a potential source for efflux has now become evident and pivotal, since methane concentrations considerably exceeding the atmospheric equilibrium value have been detected therein^12^. Sea ice- ocean coupling is given when brine rejection induced by freezing or melting generates haline convection^13^,^14^,^15^.

Tracing this process the current study reveals a unique two stage scenario: methane release from sea ice into sea water and seasonal efflux to the atmosphere. We combine oceanographic and geochemical data and highlight new information on the impact of oceanographic processes i.e. convection and haline stratification on the geochemical behaviour of methane, especially considering the pronounced seasonality resulting in the increasing decline of summer sea ice. Our studies focus on unexpected feedbacks concerning the methane cycle in the Arctic e.g. the buffering capacity of the winter mixed layer in summer and the isolation of the deeper Arctic Ocean. The permanent isolation influences the sink capacity of the Arctic Ocean which becomes relevant for methane released by terrestrial sources in the Arctic. Hence aiming on the interaction between sea ice and sea water is fundamental and has up to now not yet been considered when it comes to understanding the balancing of trace gas fluxes in the ice covered ocean. Concerning the pronounced seasonality in trace gas fluxes our observations highlight the differences between trace gas exchanges in ice-covered Polar Regions relative to open water areas in lower latitudes.

## Results

**Figure 1:**
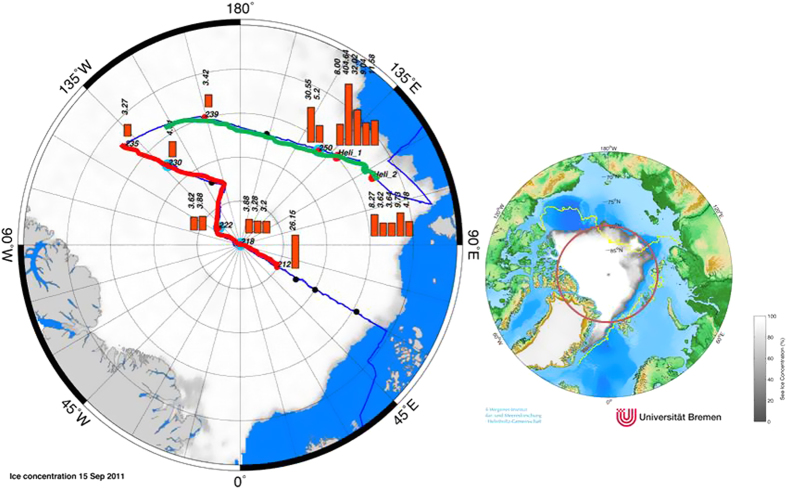
Right: Sea ice concentration in Arctic Ocean showing the minimum ice extent in 2011 (white/grey) and in 2007 (yellow line) from passive microwave satellite. The red circle encloses the region shown in Figure on the left where hydrographic transects 1 (red) and 2 (green) are displayed. Left, cruise track during R/V *Polarstern cruise* ARK-XXVI/3 (TransArc, 2011). The background image gives sea ice concentration on 15 September 2011. Dots represent sea ice stations along the track and red bars show methane concentrations (nM) in brine samples from core holes (sack holes). Methane equilibrium is 3.5 at temperature of −1 °C and salinity of 40 (see methods). Map and plots are generated with MATLAB 2013b.

The current study is based on the analysis of concentrations, stable carbon isotope ratios and aerobic oxidation rates of methane in surface water and sea ice brine coupled with the hydrographic data along two transects in the central Arctic Ocean occupied by RV Polarstern between early August and late September 2011 (Fig. 1 and methods).

### Water masses

**Figure 2:**
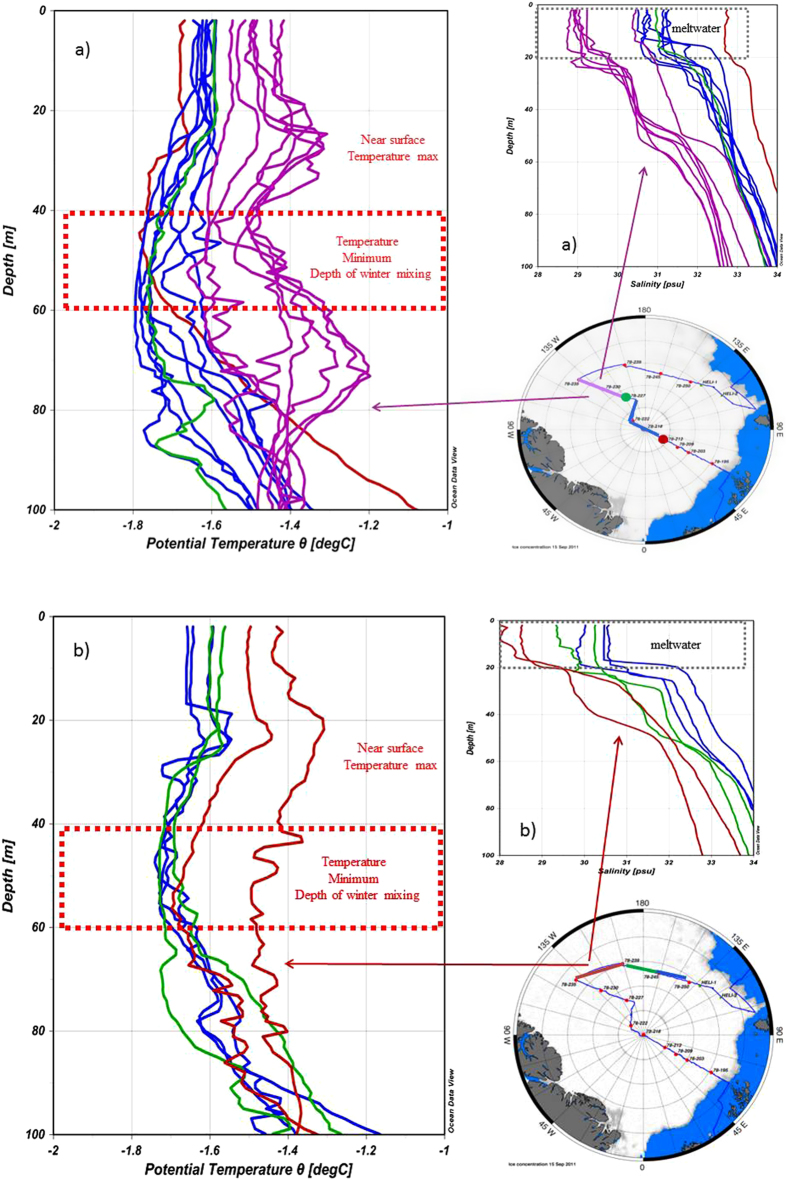
(**a**) Temperature and salinity profiles on Transect 1. Different colours represent different sections. The red dotted rectangle shows the range of the temperature minimum, i.e. the depth of winter mixing on the stations with methane excess at this depth. The temperature minimum is formed during winter when freezing induces brine rejection and haline convection but is even sustained in summer^13^,^14^,^15^. The grey dotted rectangle at the salinity profiles show the thickness of the layer, which becomes fresher by seasonal ice melt and homogenised by wind mixing (Transect 2 see Fig. 2b). Bottom right: Cruise track during R/V *Polarstern* cruise ARK-XXVI/3 (TransArc, 2011) created with JMT. The background image shows sea ice concentration on 15 September 2011. (**b**) Temperature and salinity profiles on transect 2. Different colours represent different sections. The red dotted retangle shows the range of the temperature minimum, i.e. the depth of winter mixing on the stations with methane excess at this depth. The temperature minimum is formed during winter when freezing induces brine rejection and haline convection^13^,^14^,^15^. The weaker temperature minimum on transect 2 reflects the fact that the WML is different on the two transects. The grey dotted retangle at the salinity profiles show the thickness of the layer, which becomes fresher by seasonal ice melt and homogenised by wind mixing. Bottom right: Cruise track during R/V *Polarstern* cruise ARK-XXVI/3 (TransArc, 2011) created with JMT. The background image gives sea ice concentration on 15 September 2011. Maps are generated with MATLAB 2013b.

**Figure 3:**
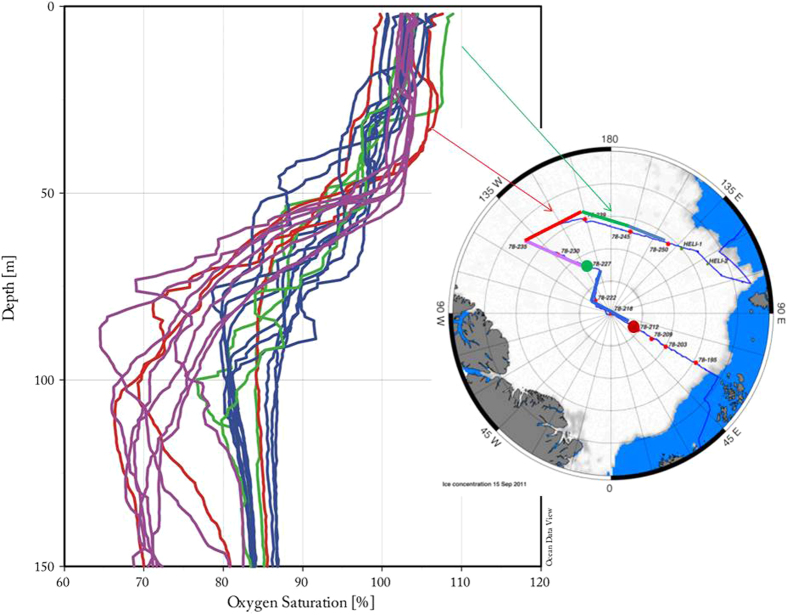
Oxygen saturation (%) as a function of depth. Clear oxygen under-saturation exists below 60 m, which is a signature of the upper halocline water (UHW)^16^. This signature is formed in the Chukchi Sea as Pacific winter water and locally formed shelf bottom water by mineralization of organic matter^16^. Left: Cruise track during R/V *Polarstern* cruise ARK-XXVI/3 (TransArc, 2011) created with JMT. The background image gives sea ice concentration on 15 September 2011. Map is generated with MATLAB 2013b.

Sea ice influenced surface water encompasses the winter mixed layer (WML) and the melt water layer (MWL). The MWL comprises the upper 20 m and is formed by melting summer sea ice (Fig. 2a,b). The WML is formed by convective overturning due to brine rejection during freezing notably with temperatures at the freezing point. This temperature minimum is even sustained in summer as warming does at many places not reach the depth of this winter mixing thus leaving a local temperature minimum and indicating regions where the WML is formed^13^,^14^,^15^ (Fig. 2a). Isolated by a strong halocline, sea water below the WML consists of upper halocline water (UHW, >60 m). The Arctic Ocean upper halocline is being created and advected from the Chukchi shelf and/or from the Bering Sea inflow and is not affected by the local surface processes^16^. The UHW is clearly detectable by the pronounced decrease in oxygen concentration (Fig. 3).

#### Methane super-saturation

**Figure 4:**
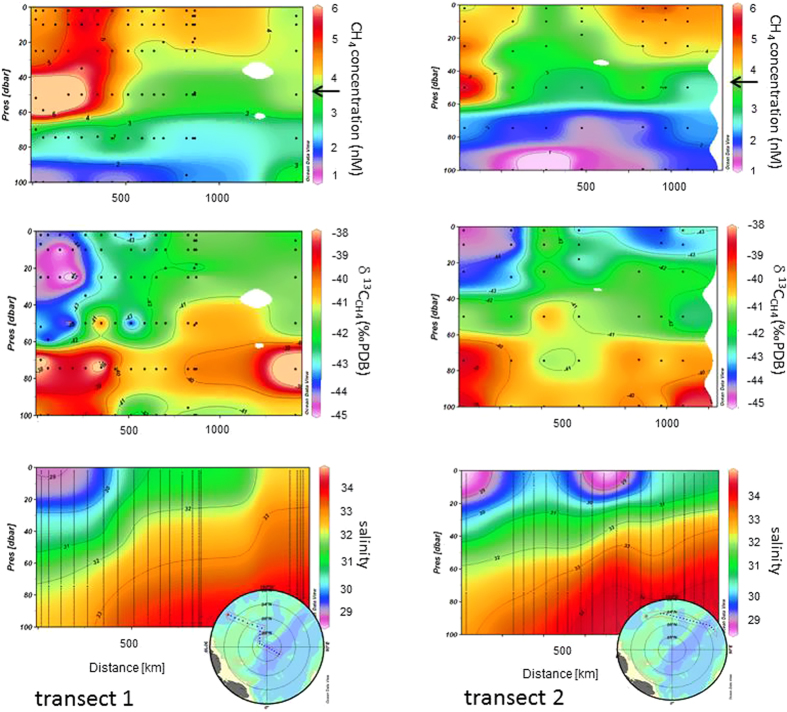
Methane concentration (nM), δ^13^C values (‰) and salinity (from top to bottom) Arrows show atmospheric equilibrium, δ^13^C value of atmospheric methane is −47‰ 17. Methane concentrations above the black arrow reflect super-saturation or under-saturation, respectively. Methane equilibrium concentration ranges between 3.7 and 3.9 nM and is calculated as a function of the gas solubility on the basis of the measured temperature (between −1.4 and –1.8 °C) and salinity (34 and 28) properties.

In the interior Arctic methane is super-saturated in the WML and MWL and under–saturated below the halocline in UHW (>60 m) (Fig. 4). We suggest that local and seasonal processes interacting with sea ice create the heterogeneous levels of methane excess. Along transect 1, methane is incorporated at the depth of the pronounced temperature minimum between 40–60 m. By comparison at transect 2 the WML contains less methane and also the temperature minimum is clearly less pronounced, indicative of lower brine rejection during winter (Figs 2b and 4). These observations indicate that sea ice may act as a methane source and brine release as the pathway to transport methane into the WML. The brine-released methane remains stored in the WML as haline stratification restricts downward mixing. Hence the methane excess in the WML is detected even in the summer while the UHW remains isolated and methane under-saturated due to the decoupling (Fig. 4). The MWL (<20 m) is less methane super-saturated than the WML. This perplexing circumstance points to the different scenarios, which create the excess in both water masses. Micro bubbles included in the brine may be entrained and then re-dissolved in the less saline seawater while the dilution with fresh water doesn’t occur in winter. In comparison methane in the MWL is released when basal melting starts but the top of the sea ice still remains impermeable inhibiting direct methane release from sea ice to the atmosphere. Then seawater flushes the brine channels and gas bubbles included in the brines will be re-dissolved. Brine super saturated with methane will then be incorporated into the MWL. However, the MWL is also diluted by fresh water from melting sea ice, thus reducing the final methane concentration therein.

#### δ^13^C signature from methane

**Figure 5:**
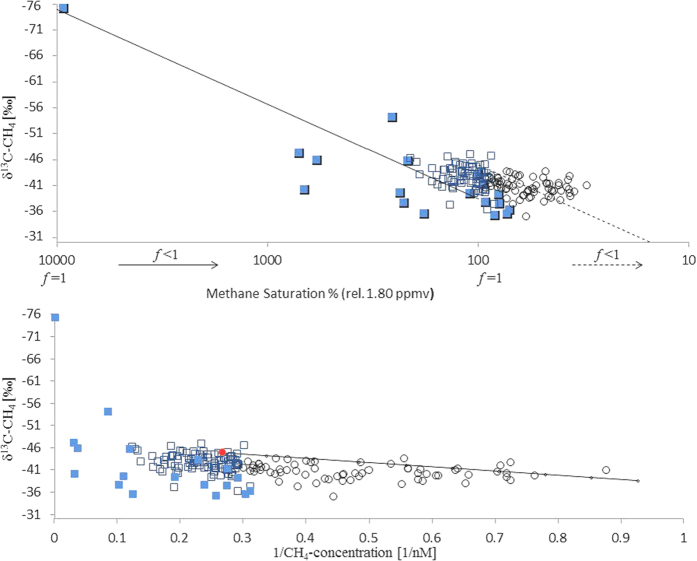
(**a**) Measured methane fitted to Rayleigh distillation curves (RC). Methane from UHW (open circles) scatter around RC1 (dashed line) indicating the δ^13^C signature resulting in consumption of initial atmospheric methane. Methane from WML and MWL (open squares) and from brine (blue squares) deviates from RC1 but is localized between RC1 and RC2 (line). RC2 results due to consumption of methane depleted in ^13^C ((δ^13^C_CH4_)_0_ −75‰), i.e. the end member of methane detected in brines (blue filled squares) (see methods). (**b**) (below): δ^13^C_CH4_ vs. the reciprocal of the methane concentration (Keeling type plot). Red dot shows the atmospheric value. Methane from UHW (open circles) scatters around a line with two endpoints: atmospheric methane and residual methane (i.e. atmospheric methane, which has been oxidized). Deviations from the mixing line reflect the influence of on-going methane oxidation. Methane from WML and MWL (open squares) and from brine samples (blue squares) extends towards higher methane concentrations (with respect to the atmospheric equilibrium) and lighter δ^13^C_CH4_ signatures than the atmospheric value and do not fit on mixing line (see methods).

The stable carbon isotopic signature of methane shows that the atmosphere and surface water in the central Arctic Ocean is not equilibrated. Deviations in the δ^13^C signature from the atmospheric background illustrate the strong evidence for the presence of additional sources of methane in surface water. We compare the measured data with calculated curves in order to establish whether the methane pool in seawater is modified by oxidation and mixing (Fig. 5).

#### A Rayleigh distillation model

i.e., δ^13^C_CH4_ = 1000* (1/α − 1)* ln *f* + (δ^13^C_CH4_)_0_ is used to calculate the δ^13^C signature of methane which results when methane is being microbially consumed (Fig. 5a). (α, the isotope fractionation factor, is 1.008^10^, *f* is the fraction of methane remaining, (1–*f*) is the methane consumed by oxidation and (δ^13^C_CH4_)_0_ is the initial isotopic composition). RC-1 (Fig. 5a, dashed line) shows the δ^13^C signature of methane that results when consumption of initial atmospheric methane, which has equilibrated in surface water, occurs. In that case (δ^13^C_CH4_)_0_ is −45‰ corresponding to the atmospheric δ^13^C_CH4_ value (−47‰)^17^ corrected by the kinetic isotopic fractionation (KIE) effect^18^. Methane residing in UHW (Fig. 5a, open circles) fits to RC-1, and indicates that the isotopic signature of this methane corresponds to that of original atmospheric methane. In comparison, methane in the winter mixed layer and melt water layer (WML and MWL, 0–60 m, blue open squares) is more depleted in ^13^C and deviates clearly from the RC-1 curve. These values are localized between RC-1 and a second Rayleigh curve (RC-2, black line, Fig. 5a), which results from consumption of methane with (δ^13^C_CH4_)_0_ −75‰, i.e. the end member of methane detected in brines (blue squares). A Rayleigh curve describes ideal conditions, assuming that only one source and only one sink (oxidation) exists. The measured data have been compared with calculated curves in order to check if the methane pool in seawater is modified by oxidation. Deviations of the measured values from the calculated Rayleigh curves reflect the influence of additional processes, i.e. mixing with sources with different isotope signatures and sea-air flux. Since not only oxidation but a combination of several processes modifies the isotopic signature of methane in sea water we also displayed our data using a Keeling type plot.

#### 

A Keeling type plot is used (Fig. 5b) to calculate the mixing between two assumed end-members. The calculated mixing trend is compared with the measured values to illustrate potential mixing of methane of different isotopic signatures. Mixing end points are: atmospheric methane and residual methane. Residual methane is the pool of initial atmospheric methane, which has been oxidised up to the threshold value where microbial consumption finally ceases because of substrate limitation. Methane residing in UHW scatters around the mixing line between atmospheric and residual methane as end members and exerts an important control on the distribution of the isotopic ratio of methane from these precursors. In comparison methane residing in the WML and MWL does not fit the mixing line, which is evidence for the presence of additional sources.

## Discussion

Along both transects we observed distinct lateral and vertical variations both in concentration and in the δ^13^C signature of methane and suggest that the surface Arctic Ocean is not equilibrated with the atmosphere. Methane is commonly super-saturated down to the WML. Being isolated by a strong salinity gradient the underlying water masses are homogeneously under-saturated with respect to the atmospheric equilibrium concentration (Fig. 4). They mainly consist of upper halocline water (UHW) as indicated by the low oxygen saturation^16^ (Fig. 3). This signature is formed in the Chukchi Sea. Consequently, a lateral transport of methane in sea water from the shelf areas into the central Arctic can be ruled out.

Compared to the atmospheric background^17^, methane in sea water is enriched in ^13^C. The preferred microbial use of ^12^C makes methane consumption the most likely cause of this isotopic shift^19^. In addition mixing of methane of different isotopic signatures from other sources may contribute to deviations from the background signal. Hence, to trace the isotopic fractionation, which potentially occurs when methane with different initial δ^13^C signatures is being consumed or mixed, we calculated a Rayleigh distillation and Keeling type plot (Fig. 5). Differences between methane residing in UHW and methane residing in sea ice-influenced water (i.e. WML and MWL) become obvious by comparing the measured values with calculated curves. Methane residing in UHW scatters around an oxidation line and a mixing line between atmospheric and residual methane (i.e. threshold value) as end members and exerts an important control on the distribution of methane from these precursors. Decoupling from the atmosphere and simultaneous long-term methane consumption creates this pool of under-saturated and ^13^C enriched methane. In comparison, methane from sea ice-influenced water (i.e. WML and MWL) is more depleted in ^13^C than methane in UHW and values scatter around an oxidation line with methane from brine as end member (Figs 4 and 5).

The large potential of sea ice to discharge methane is evident in methane super-saturation of up to 8000% in brine (Fig. 1). Furthermore, considerable alterations in the δ^13^C signature (−36‰ to −75‰) of methane in brine are indicative of extensive methane cycling within sea ice i.e. production and consumption, as both processes induce isotopic fractionation. Oxygen depletion in brine channels^20^ and microbial production of up to 300 mg C mg^2^ d^−1^ 21 favour anaerobic degradation of organic matter and are the most likely preconditions for methane production in sea ice.

Methane in sea ice may be present in two phases; dissolved in brine or most likely in small gas bubbles formed during freezing. Temperature and salinity changes lead to a sharp decrease in methane solubility, in which case a much larger amount of methane will accumulate as gas in the ice^12^,^22^. In winter, sea ice is to a large extent impermeable and the ice-air gas transfer to the atmosphere becomes severely restricted^12^,^23^. Then the discharge of methane dissolved in brine becomes relevant. Also micro-bubbles included in the brine may be entrained and then re-dissolved in the less saline sea water. The depth of winter mixing by brine rejection is clearly indicated by the temperature minimum even in the following summer^13^,^14^,^15^ (Fig. 2). Hence we conclude that methane ejected with brine during winter becomes dispersed in the WML and may remain stored until the following summer (Fig. 4).

**Figure 6:**
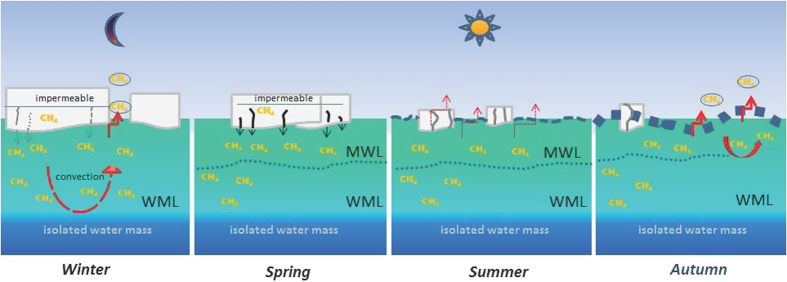
Seasonal differences in methane cycling between sea ice, surface water and atmosphere. In **winter** deep convection transports brine, charged with methane, into the WML. Methane efflux may occur by convection in leads, while efflux through impermeable sea ice is restricted. In **spring**, basal melting starts and transports methane (dissolved or re-dissolved gas bubbles) into the MWL. In **summer**, stabilized by thermal stratification and less turbulence, methane remains entrained in both layers. In **autumn**, sea surface temperatures drop, enabling methane efflux by surface water convection. Water masses below the WML are not affected by this methane cycling.

In spring, methane in the ice may be released to the MWL, when basal melting starts but the top of the sea ice still remains impermeable, inhibiting direct methane release from sea ice to the atmosphere. Subsequently, sea water or melt water will flush the brine channels and gas bubbles included in the brines will re-dissolve. Several freezing and melting events may contribute to locally different levels of super saturation in the MWL (Figs 4 and 6).

In summer, the methane excess remains preserved, as on the one hand, haline stratification restricts downward mixing and on the other, the reduced turbulence and restricted gas transfer in the presence of sea ice^8^,^24^ impedes the efflux (Fig. 6). Excess methane is also conserved by slow methane consumption. Seawater sampled directly under the ice shows methane oxidation rates at the lower end of reported data (0–820 nM/d)^25^, i.e. rates with a median 0.013 nM/d using a ^3^H-CH_4_ tracer and with a median of 0.0009 nM/d with a ^14^C-CH_4_ tracer, respectively. Although both tracers increase ambient methane concentrations by 1.7 nM and 457 nM, respectively, an increase in uptake rate due to an increase in substrate was not observed over three days incubation. These pulse chase experiments illustrate that the abundance and activity of methanotrophic bacteria is low in the water investigated. Methanotrophs might be limited due to unfavourable growth conditions (e.g., low temperature or lack of copper^26^,^27^) since only bacteria with a low uptake velocity inhabit surface water. A weakly developed methanotrophic community due to restricted mixing with other water masses could also prevent the seeding with methanotrophic bacteria^28^.

In autumn, cooling and freezing generate convective mixing down to the halocline that will both homogenize methane excess in surface sea water and initiate methane efflux allowing the cold water to equilibrate with the atmosphere. After a new sea ice cover has formed and methane super saturation is still present, methane efflux may occur from leads or fractional sea ice cover during the entire winter (Figs 4 and 6).

The methane surplus in sea ice-influenced water (i. e. WML and MWL) ranges between 1.5 and 1.9 nM relative to the methane equilibrium concentration (see methods). By extrapolating the methane surplus in the top 10 m to the area of seasonal sea-ice melting (6.4 million km^2^), we calculate that between 7.5 and 10 Gg of methane may be stored in sea ice during summer. This estimate should be considered as being on the low side, since only the surplus methane near the surface is taken into account. Methane stored in sea ice will exceed this amount considerably but to confirm this, more measurements are needed. Summer sea ice retreat may change the amount of excess methane eventually making estimates of future atmospheric emissions more uncertain. In addition, increasing summer sea ice retreat strengthens the density stratification in the upper ocean and consequently the decoupling of sea ice- influenced water masses from the UHW. The permanent isolation of the UHW from the atmosphere becomes evident by the homogeneous methane under-saturation (Figs 4 and 6). Several years are needed for UHW to cross the Arctic Basin^29^. During that long journey on-going microbial methane consumption, albeit slow, creates isotopic fractionation, which eventually enriches the residual methane in ^13^C. Thus not only does the concentration steadily decrease during the long-term water mass separation, but methane also becomes heavier (Fig. 4). We found that methane is consumed down to a concentration, which corresponds to less than 50% of the saturation level. A depletion of this magnitude results in threshold values, i.e. values where consumption finally ceases because of substrate limitation^30^. The depth, at which threshold values is reached differs slightly along transects, i.e. between Atlantic-derived and Pacific-derived water and depends on and corresponds to the depth of haline winter convection. The permanent isolation of the UHW reduces the capacity of the interior Arctic Ocean to act as a sink for increasing atmospheric methane. This decoupling from the deeper ocean reinforces the near surface cycling of methane (i.e. between sea ice-influenced water, sea ice and the atmosphere). Summer sea ice retreat triggers this pathway with hitherto unknown consequences for the source as well as sink capacity for this greenhouse gas in the Arctic.

## Methods

### Sampling

We measured concentrations, stable carbon isotope ratios and aerobic oxidation rates of methane in surface water and sea ice brine along two hydrographic transects in the central Arctic Ocean occupied by RV Polarstern between early August and late September 2011. The first transect runs from 82.5°N, 50^o^ E via the North Pole to the Alpha Ridge, the second transect runs along 84°N over the Makarov Basin into the Amundsen Basin (Fig. 1). Covering both Atlantic and Pacific derived water, our sampling was carried out in regions with different ice coverage. We use hydrographic data (salinity, temperature and oxygen) and water samples down to 100 m depth obtained with a ship-borne CTD (Seabird) and a mounted Rosette. **Salinity and temperature** were measured with a Seabird SBE 911 plus CTD. Oxygen was measured with the SBE 43 dissolved oxygen sensor SN 743 and sensor calibration was done on water samples using Winkler titration. Water samples from up to six different depths were collected during the upcast at each CTD station with 10 L Niskin bottles mounted on a rosette sampler. Sea ice brine was collected by extracting ice cores from sea ice to depths of up to 50 cm and not more than half way through the ice depth, using a KOVACS Mark II ice corer. The remaining, so called “sack holes” were covered and left to allow brine to drain into the holes for a few minutes, after which brine was removed with a syringe and treated the same way as water samples. **Methane concentrations** were analysed within a few hours after sampling. The dissolved gas was extracted from the water or brine by vacuum-ultrasonic treatment and subsequently measured with a gas chromatograph (Chrompack 9003 (GC) with a flame ionization detector (FID). For gas chromatographic separation we used a packed column (Porapac Q 80/100 mesh). The GC oven was operated isothermally (60 °C) and the FID was held at 250 °C. Two sets of standard gas mixtures were used for calibration. The standard deviation of duplicate analyses was 5%. This high overall error is almost exclusively due to the gas extraction procedure and not to GC precision, which had an error of only 1%. After GC analyses an aliquot of the extracted gas was transferred into pre-evacuated glass containers for analysis of the stable carbon isotopic signature on shore.

The **δ**^**13**^**C**_**CH4**_ **values** were determined using a Delta XP plus Finnigan mass spectrometer. The extracted gas was purged and trapped with PreCon equipment (Finnigan) to pre-concentrate the sample. All isotopic ratios were given in a δ-notation relative to the Vienna Pee Dee Belemnite (VPDB) standard using conventional delta notation. The reproducibility derived from duplicates was 1–1.5‰.

A significant correlation between 1/methane concentration vs. the δ^13^C values (R^2^ = −0.84) is given for methane in UHW when y- intercept is −47‰. In sea ice-influenced water (i.e. WML and MWL) a correlation is missing when y- intercept is −47‰ (R^2^ = 0.0554) but given when the y- intercept is −55‰. This different correlation refers to deviating methane sources in both water masses.

### Methane consumption

Water was sampled directly from the ice floes through ice core holes for *ex situ* tracer incubations in order to quantify the methane consumption. Methane oxidation rates were determined from *ex situ* incubations of water samples in 100 ml serum vials. Samples were incubated with 50 μl gas mixture comprised of ^3^H-labelled CH_4_ (200–300 Bq) and a second set was incubated with 10 μl of ^14^C-labelled CH_4_ (12000–15000 Bq). The samples were subsequently shaken to facilitate tracer dissolution and then incubated in the dark at 0 °C. Tracer consumption was measured after 48 h and 72 h. Incubations with ^14^C-CH_4_ were terminated by adding a 5 ml headspace and injecting 0.5 ml of 10 M NaOH so that the remaining ^14^C-CH_4_ accumulated in the headspace and the produced ^14^C-CO_3_^2-^ and ^14^C-biomass was trapped in the aqueous NaOH solution. Separation and activity measurement of ^14^C-CH_4_ and ^14^C-CO_3_^2−^ was carried out analogous to previous measurements of CH_4_ turnover in sediments^25^ (and references therein). In short, ^14^C-CH_4_ in the headspace was combusted to ^14^C-CO_2_, while ^14^C-CO_3_^2−^ was converted to ^14^C-CO_2_ through acidification with HCl. In either case, ^14^C-CO_2_ was then trapped and the radioactivity was measured by wet scintillation counting. Incubations with ^3^H-CH_4_: Total activity (^3^H-CH_4_ + ^3^H-H_2_O) was measured in 1 ml of sample aliquot by wet scintillation counting and activity of ^3^H-H_2_O was measured after sparging the sample for ≥30 min with nitrogen gas to remove remaining ^3^H-CH_4_. CH_4_ oxidation rates (*r*_OX_) were calculated assuming first order kinetics: r_OX_ = *k*’ [CH_4_] where *k*’ is the effective first order rate constant calculated as the fraction of labelled CH_4_ oxidized per unit time and [CH_4_] is the ambient CH_4_ concentration. Killed controls resulted in methane oxidation rates near zero.

## Additional Information

**How to cite this article**: Damm, E. *et al.* Methane excess in Arctic surface water-triggered by sea ice formation and melting. *Sci. Rep.* **5**, 16179; doi: 10.1038/srep16179 (2015).
